# Giant cell myocarditis causing sudden death in a patient with sarcoidosis

**DOI:** 10.4322/acr.2020.238

**Published:** 2020-11-20

**Authors:** John P. Ziegler, Nicholas I. Batalis, James W. Fulcher, Michael E. Ward

**Affiliations:** 1 Medical University of South Carolina, Charleston, SC, USA; 2 Medical University of South Carolina, Department of Pathology, Charleston, SC, USA; 3 District 7 Medical Examiner, Volusia County, FL, USA; 4 University of South Carolina School of Medicine Greenville, Greenville, SC, USA; 5 Office of the Medical Examiner, Greenville County, SC, USA

**Keywords:** Myocarditis, Sarcoidosis, Death, Sudden, Cardiac, Autoimmune Diseases, Case Reports

## Abstract

Giant cell myocarditis (GCM) is a rare and rapidly fatal cardiovascular condition most often seen in young adults. It is characterized microscopically by myocardial necrosis with multinucleated giant cells in the absence of well-defined granulomas. This disorder has typically been attributed to manifest as heart failure, but in some individuals, GCM may present as sudden cardiac death. Herein, we present a fatal case of GCM in a 36-year-old male with a history of autoimmune disorders. The decedent presented to the emergency room due to vomiting and was treated for nausea due to suspected dehydration. He was discharged that night and found dead on his bathroom floor the following day. Postmortem examination revealed psoriasis and granulomatous lesions in the lungs consistent with sarcoidosis, further supporting circumstantial evidence existing between GCM and autoimmune disorders. Additionally, this case provides an opportunity to distinguish GCM from the distinct clinical entity of cardiac sarcoidosis (CS), especially in the setting of systemic sarcoidosis. We hope to raise awareness of this rare disease process and its potential to cause sudden cardiac death so that it may be considered in a differential diagnosis as immunosuppression and early cardiac transplantation largely determine the prognosis.

## INTRODUCTION

Giant cell myocarditis (GCM), commonly referred to as idiopathic giant cell myocarditis (IGCM), is a rare disorder that typically affects young to middle-aged adults that was first described in 1905 as a mixed myocardial infiltrate with multinucleated giant cells and cardiomyocyte necrosis.^1^ This definition has since been updated to distinguish GCM from the distinct histological entity of cardiac sarcoidosis (CS). In 1956, Tesluk^2^ differentiated the diffuse nongranulomatous infiltrate of GCM from the well-organized granulomatous lesions of CS. These poorly formed granulomas are still considered characteristic of GCM, with structured, follicular granulomas excluding it by definition.^3^

Giant cell myocarditis is extremely rare with less than 300 reported cases since its initial distinction from myocarditis in 1905,^4^ though this figure is likely a gross underestimation of the true incidence as the majority of patients dying from GCM do not undergo autopsy.^5^ Giant cell myocarditis is a disease characterized by rapid deterioration in ventricular function, frequent ventricular arrhythmias, and complete heart block. It has a swift disease course with often only three weeks between onset symptoms and hospital presentation.^6^ Congestive heart failure, fatal ventricular dysrhythmias, and sudden cardiac death (SCD) are common outcomes if not treated early.^6^ ^,^ ^7^ This grim prognosis and fulminant disease course illustrate the importance of awareness and detection of this disease to potentially avoid fatal outcomes.

Herein, we present a case of SCD due to GCM in a patient also found to have probable pulmonary sarcoidosis with the goal of increasing our understanding of the defining characteristics, presentation, distinction, and etiology of GCM along with exploring the relationship of GCM with sarcoidosis and other autoimmune diseases.

## CASE REPORT

A 36-year-old white male who reported not feeling well was seen at the local emergency room for nausea and vomiting due to suspected dehydration. He was given intravenous fluids, ondansetron, and metoclopramide and then deemed stable for discharge. He was also prescribed promethazine for nausea and was instructed to continue taking his home medications, which included halobetasol, lisdexamfetamine, and sertraline. He was found unresponsive on his bathroom floor by his mother the following afternoon and death was pronounced that evening. The autopsy was performed the following morning. Access to previous encounters was not available.

## AUTOPSY PRESENTATION

The decedent’s external examination was largely unremarkable, showing a well-developed, well-nourished, adult Caucasian male who weighed 75.8 kilograms and was 1.70 meters in height. Pink, scaly skin covered the extensor surfaces of his upper and lower extremities, consistent with his clinical history of psoriasis.

**Figure 1:**
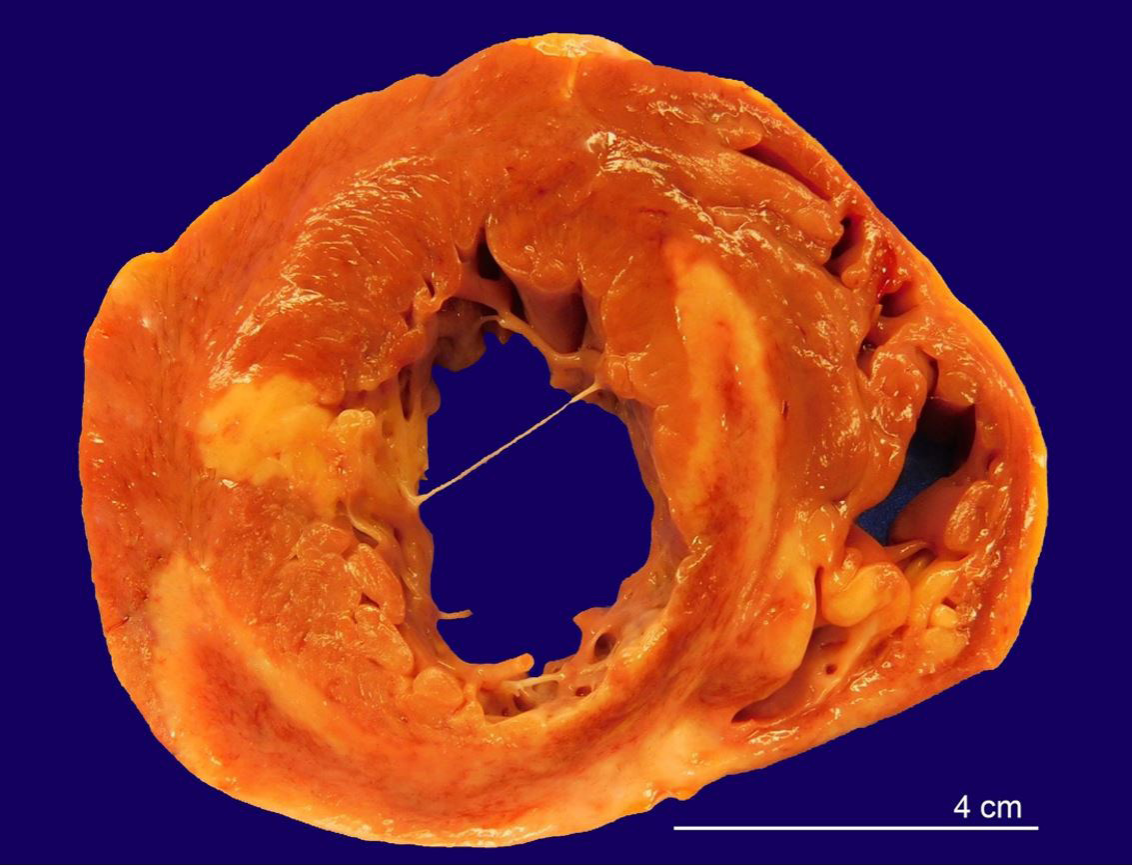
Cross-section of heart showing multifocal areas of yellow to pink-tan necrotic discoloration throughout the ventricular myocardium.

The heart weighed 510 grams (mean reference range [mRR]; 327 g) and was otherwise unremarkable externally. Upon sectioning, the myocardium was dark, red-brown, and firm with multiple areas of yellow-tan discoloration involving both ventricles and extending from apex to base (Figure 1). These areas ranged from 0.7 to 3 cm and were partial to full-thickness in distribution.

**Figure 2:**
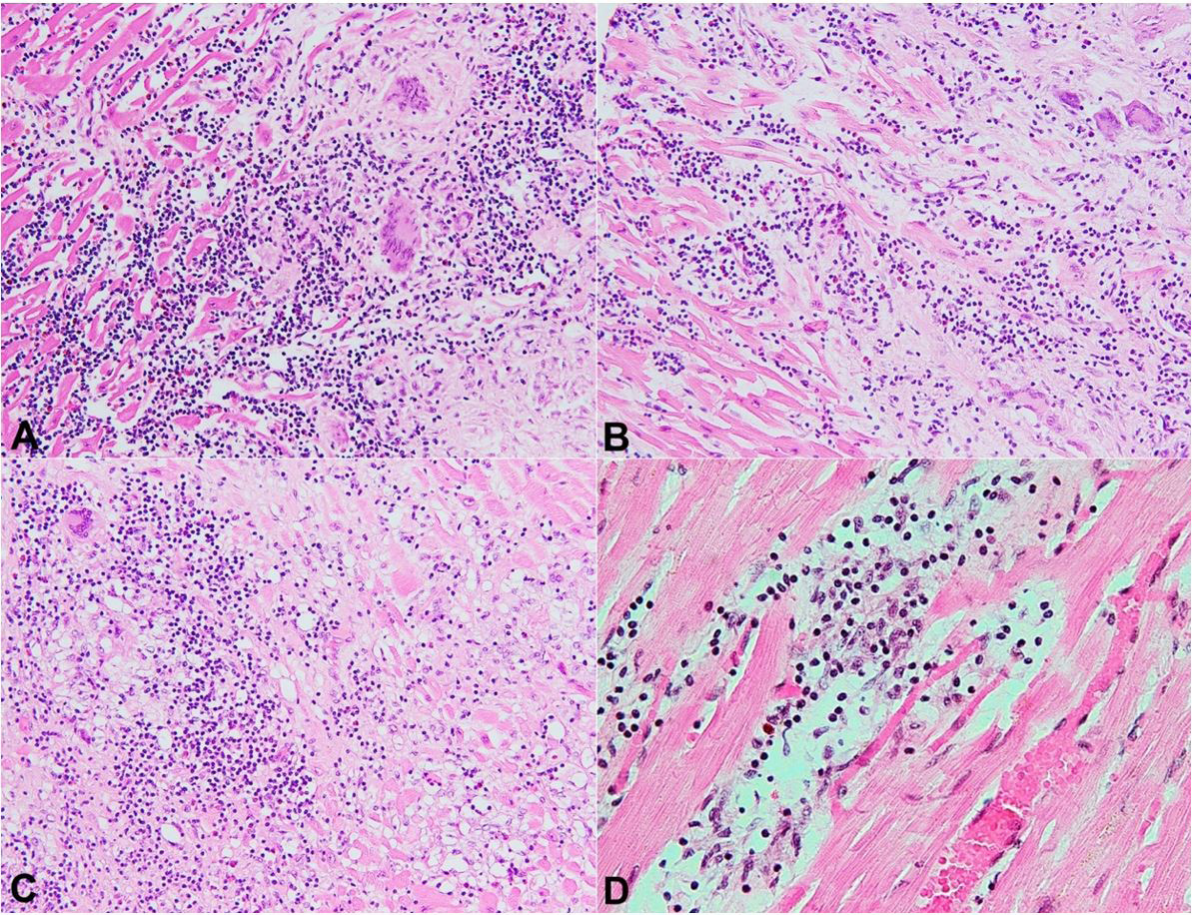
**A –** Histologic section of leading edge of heart lesion showing a mixed inflammatory infiltrate with prominent eosinophils, multinucleate giant cells, and myocyte necrosis (H&E, 200X); **B –** Another histologic section showing mixed inflammation with giant cells and myocyte injury (H&E, 200X); **C –** Histologic section showing mixed inflammation with giant cells and widespread myocyte injury with vacuolization (H&E, 200X); **D –** Histologic section away from large areas of necrosis with mixed inflammation and myocyte injury in the absence of giant cells (H&E, 200X).

Three sections were taken for microscopic examination, and revealed geographic replacement of myocardium by confluent areas of central fibrosis and necrosis with scattered multinucleate giant cells and a leading edge with a mixed inflammatory infiltrate with lymphocytes, plasma cells, eosinophils, and giant cells with associated myocyte necrosis (Figures 222C). Additionally, there were some foci of nearby inflammation and myocyte necrosis in the absence of giant cells (Figure 2D). Sections of myocyte loss were seen extensively in the left ventricle and also in the right ventricular myocardium. No distinct, well-formed granulomas were noted. Moderate atherosclerotic coronary vascular disease was appreciated. Cardiomegaly was likely secondary to cardiac insufficiency in combination with increased organ stress due to giant cell deposits inhibiting cardiac contractility.

The right and left lungs weighed 790 and 830 grams respectively (mRR; 450g and 375 g respectively) and were found to be edematous grossly. The upper and lower airways were patent, smooth, yellow-tan and largely unremarkable. There were notable focal dense fibrous adhesions of the left lung to the lateral chest wall adjacent to an old surgical site.

**Figure 3:**
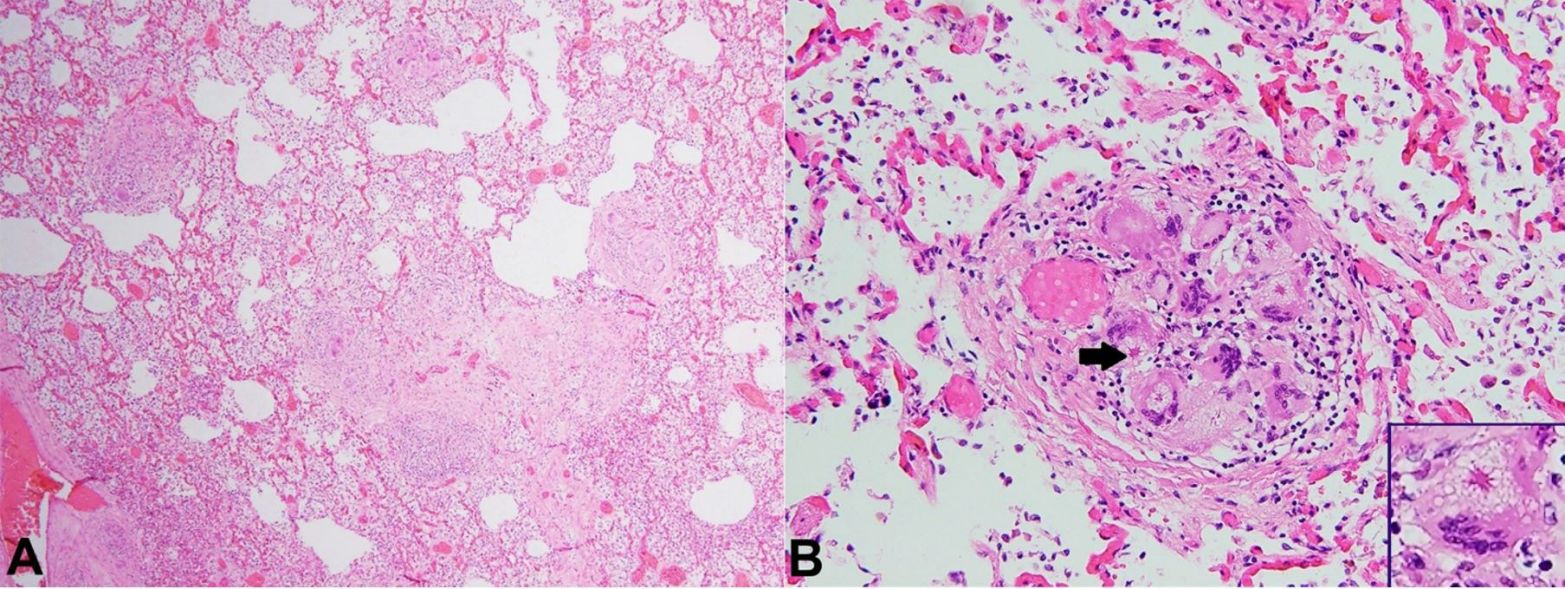
Histologic sections of lung showing: **A –** Scattered noncaseating, “naked” granulomas throughout the pulmonary parenchyma (H&E, 40X); **B –** High-power view of a single granuloma showing multiple giant cells and asteroid bodies (arrow and inset) (H&E, 400x).

Three microscopic sections were submitted and showed scattered, well-defined, non-caseating granulomas with minimal associated inflammation throughout the parenchyma, some containing prominent asteroid bodies (Figure 3).

These granulomas were present within the interstitium and were both peribronchial and perivascular in distribution and consistent with pulmonary sarcoidosis. Bilateral hilar lymphadenopathy was appreciated.

Other pathologies considered in the differential diagnosis for the pulmonary granulomas included mycobacterium tuberculosis, histoplasmosis, blastomycosis, bronchocentric granulomatosis, hypersensitivity pneumonitis, pneumoconiosis, foreign body granulomatosis, granulomatosis with polyangiitis, and eosinophilic granulomatosis with polyangiitis, but, no history of recent travel to endemic areas, environmental or occupational exposures, prior illnesses, IV drug use, or evidence of vasculitides make these etiologies less likely. The patient’s lack of systemic symptoms such as fever, night sweats, and weight loss also decreased concern for infection or malignancy. No foreign material was identified under polarized light and specialized stains for mycobacterium and fungal organisms were not deemed necessary. Hypersensitivity pneumonitis (HP) was given additional consideration, but HP granulomas are typically smaller, not discrete, and are associated with greater interstitial chronic inflammation. These characteristics were inconsistent with our findings.

Gross examination of the other organs was unremarkable. The liver was enlarged and weighed 2240 grams (mRR; 1980 g), but there was no significant edema or cavity effusions. Single histopathologic sections were also taken from the liver, kidney, and brain. The liver section showed mild to moderate sinusoidal dilation and congestion but no necrosis or granulomas. No histopathological abnormalities were noted in the kidney and brain; the spinal cord was not examined. Toxicology of femoral blood was only positive for tetrahydrocannabinol (THC). The cause of death was determined to be sudden cardiac dysrhythmia due to giant cell myocarditis with a secondary post-mortem discovery of probable pulmonary sarcoidosis.

## DISCUSSION

This case contains characteristics typical of GCM but raises interesting questions concerning the defining attributes of GCM, its distinction and possible relationship to sarcoidosis, and overall relation to autoimmune disease. Search criteria on PubMed included “giant cell myocarditis” AND sarcoidosis and yielded 40 results. 34 papers were omitted by the authors, resulting in six included papers describing GCM in the setting of sarcoidosis

### Incidence and Demographics

Giant cell myocarditis is a rare pathologic entity that has been reported to occur in 0.007-067% of populations across the globe.^7^ ^-^ ^9^ Cases tend to have an equal gender distribution and the median reported age is approximately 42 years (range 10 days- 88 years).^6^ ^,^ ^10^ ^-^ ^12^ It should be noted, though, that there is likely a gross underestimation of the true incidence as 60% of cases are diagnosed at transplant or autopsy, and these procedures are not routinely performed on an unselected population.^4^ ^,^ ^5^

### Microscopic Features of Giant Cell Myocarditis and Distinction from Cardiac Sarcoidosis

Davies et al.’s^13^ study distinguished this form of myocarditis by the formation of giant cells from myocardial cells, much like regeneration of skeletal muscle following an injury. In 1995, another study supported a monocytic-histiocytic lineage as the well-developed endoplasmic reticulum and lysosomal vacuoles were characteristic of monocytes and macrophages.^14^ A recent study^5^ has recognized giant cells as CD68þ macrophages that are usually at the border of active inflammation surrounding the necrotic area. It has also been found that CD4+ T cells are the principal infiltrating lymphocyte in GCM.^15^ The classification of giant cells as macrophages and presence of pervading T lymphocytes support a theory of immune dysregulation mediated by T lymphocytes, which is further supported by presence of nonspecific antisarcolemma and antimyosin antibodies.^16^ In addition to giant cells and lymphocytes, GCM is typified by an aggressive, mixed inflammatory infiltrate, often with abundant eosinophils.

To further grasp GCM’s complex pathology, it must be differentiated from the similar-appearing cardiac sarcoidosis (CS), which depends solely on the histopathology of the myocardium.^17^ Tesluk^2^ differentiated the diffuse, nongranulomatous infiltrate of GCM from the well-organized, granulomatous lesions of CS. Cardiac sarcoidosis has been further defined by noncaseating, interstitial granulomas without myocyte necrosis.^18^ As can be seen from the literature above, it is the presence of a sarcoid-like granuloma that serves as the differentiating characteristic between the two diseases; yet, GCM can often still be the prime manifestation of a systemic granulomatous process. Sarcoidosis or granulomatous infiltration of other organs does not exclude diagnosis of GCM.^3^ This clarification is essential as the decedent in this case was given a post-mortem diagnosis of pulmonary sarcoidosis, an inflammatory disease defined by the presence of granulomas on the lungs. These co-existing diseases support GCM as a systemic process of immune dysregulation.

Additionally, Okura et al.’s^11^ study distinguished GCM as a separate entity from CS. It compared a cohort of 73 GCM patients to a registry consisting of 42 patients with CS. In the initial microscopic examination, CS specimens presented with increased histological presence of granulomas and fibrosis while the GCM specimens had more necrosis, foci resembling lymphocytic myocarditis, and eosinophils.

### Presenting Symptoms and Mechanism of Death in Giant Cell Myocarditis

GCM has diverse presenting symptoms seen throughout the literature including dyspnea, fatigue, palpitations, chest pain, hypotension, headache, runny nose, and myalgias among others.^16^ ^,^ ^19^ Other increasingly serious symptoms include rapidly progressive heart failure, acute coronary syndromes, dysrhythmias, heart block, severe left ventricular dysfunction with cardiac tamponade or cardiogenic shock, or even sudden death.^6^ ^,^ ^9^ ^,^ ^15^ ^,^ ^16^ This extensive list of symptoms and conditions poses a significant obstacle that may delay a time-crucial diagnosis. The decedent in this case presented with only nausea and vomiting on the day prior to his death. Although uncommon expressions of the illness, nausea and vomiting have been documented previously in the literature.^19^

Clinically, GCM and CS usually distinguish themselves quite clearly. Although congestive heart failure, ventricular arrhythmias, and heart block have been associated with both CS and GCM,^6^ patients with GCM are more likely to present with a fulminant course with a shorter period from symptom onset to death or transplantation than CS. In one study, the transplant-free survival probability was 10% in the GCM group and 60.5% in the CS group five years after symptom onset.^11^ Giant cell myocarditis features a grim prognosis, but improved immunosuppressive treatments have been successful in reducing inflammation and postponing the time until transplantation or death.^5^ ^-^ ^7^ ^,^ ^10^ ^,^ ^17^

Cooper et al.’s^6^ hallmark study and registry established GCM’s gloomy prognosis in which 89% of patients either died or underwent transplantation with a median transplant-free survival time of only 5.5 months from symptom onset if left untreated. The study also determined the leading mechanism of death to be congestive heart failure (75%). Ventricular dysrhythmia (14%) and heart block (5%) were the next prominent mechanisms. Consequently, heart failure has been unequivocally cited as the leading mechanism of death for nearly 20 years. In 2016, Ekstrom et al.^7^ challenged these findings by creating the largest single-center population of GCM with 51 patients spanning from 1991 to 2016. This study found sudden ventricular dysrhythmias to be a more common mechanism of death than heart failure. The study also discovered that SCD, as seen in this decedent, was a much more common mechanism in non-transplanted GCM than was terminal heart failure.

### Etiology - Autoimmunity

Throughout the literature, GCM has been commonly associated with or suggested to be an autoimmune disorder.^6^ ^,^ ^9^ ^-^ ^11^ ^,^ ^13^ ^,^ ^15^ ^,^ ^16^ ^,^ ^18^ ^,^ ^20^ ^-^ ^22^ Specific associations with autoimmune diseases such as ulcerative colitis, Crohn disease, Hashimoto thyroiditis, rheumatoid arthritis, pernicious anemia, and others have been the grounds by which the majority of authors have made their presumptions. Overall, Cooper et al.’s^6^ study found 19% of GCM patients had associated autoimmune diseases.

Perhaps the most influential piece of evidence supporting an autoimmune etiology of GCM was an animal study in 1990.^20^ Researchers were able to induce GCM in rats using autoimmunization with myosin. This technique solidified autoimmunity as a highly feasible pathway for inducing GCM in a host. Further, GCM has been considered the prime manifestation of a systemic granulomatous process.^3^ These granulomas most often exist in lungs, liver, lymph nodes, and spleen.^9^ ^,^ ^13^ ^,^ ^23^ For this reason, granulomas suggest that at least some cases of GCM are part of a systemic autoimmune disease rather than an isolated organ-specific autoimmune reaction.^9^

The decedent in this case presented with psoriasis and had the post-mortem diagnosis of probable pulmonary sarcoidosis, two diseases commonly considered to be autoimmune, further supporting the relationship between GCM and autoimmunity.

## CONCLUSION

Giant cell myocarditis involves a mixed inflammatory infiltrate of the heart with prominent giant cells and myocyte necrosis. The disease manifests itself most often as a ventricular dysrhythmia, congestive heart failure, or sudden cardiac death but presents with a wide variety of other symptoms. The etiology of the disease has not been officially established but many have noted GCM’s autoimmune nature, so this diagnosis must be considered in patients with pre-existing autoimmune diseases, including sarcoidosis, as seen in this case. Additionally, the presence of sarcoidosis in other organs does not preclude a diagnosis of GCM.

The decedent’s cause of death was giant cell myocarditis manifesting as sudden cardiac death. His symptoms were atypical, presenting only with nausea and vomiting the day prior to his death. The history of psoriasis and post-mortem diagnosis of probable pulmonary sarcoidosis revealed an association with autoimmune disorders, a characteristic seen in about 20% of reported cases of GCM. This case demonstrates a unique and rapid manifestation of GCM and raises interesting questions concerning the future research, treatment course, and complex etiology of the disease.

## References

[1] Saltykow S (1905). Über diffuse myokarditis. Virchows Arch Pathol Anat Physiol Klin Med.

[2] Tesluk H (1956). Giant cell versus granulomatous myocarditis. Am J Clin Pathol.

[3] Cooper LT (2000). Giant cell myocarditis: diagnosis and Treatment. Herz.

[4] Cooper LT, Elamm C (2012). Giant cell myocarditis: diagnosis and treatment. Herz.

[5] Xu J, Brooks EG (2016). Giant cell myocarditis: a brief review. Arch Pathol Lab Med.

[6] Cooper LT, Berry GJ, Shabetai R (1997). Idiopathic giant-cell myocarditis: natural history and treatment. N Engl J Med.

[7] Ekström K, Lehtonen J, Kandolin R, Räisänen-Sokolowski A, Salmenkivi K, Kupari M (2016). Incidence, risk factors, and outcome of life-threatening ventricular arrhythmias in giant cell myocarditis. Circ Arrhythm Electrophysiol.

[8] Wakafuji S, Okada R (1986). Twenty year autopsy statistics of myocarditis incidence in Japan. Jpn Circ J.

[9] Vaideeswar P, Cooper LT (2013). Giant cell myocarditis: clinical and pathological features in an Indian population. Cardiovasc Pathol.

[10] Rosenstein ED, Zucker MJ, Kramer N (2000). Giant cell myocarditis: most fatal of autoimmune diseases. Semin Arthritis Rheum.

[11] Okura Y, Dec G, Hare JM (2003). A clinical and histopathologic comparison of cardiac sarcoidosis and idiopathic giant cell myocarditis. J Am Coll Cardiol.

[12] Özdemir-Kara D, Pehlivan S, Türkkan D, Alkan-Alkurt H, Akduman B, Karapirli M (2016). Idiopathic giant cell myocarditis in a newborn: case report. Turk J Pediatr.

[13] Davies MJ, Pomerance A, Teare RD (1975). Idiopathic giant cell myocarditis: a distinctive clinico-pathological entity. Br Heart J.

[14] Cooper LT, Berry GJ, Rizeq M, Schroeder JS (1995). Giant cell myocarditis. J Heart Lung Transplant.

[15] Kumari MK, Mysorekar VV, Praveen S (2012). Idiopathic giant cell myocarditis: a case report. J Clin Diagn Res.

[16] Koul D, Kanwar M, Jefic D (2010). Fulminant giant cell myocarditis and cardiogenic shock: an unusual presentation of malignant thymoma. Cardiol Res Pract.

[17] Kandolin R, Lehtonen J, Salmenkivi K, Räisänen-Sokolowski A, Lommi J, Kupari M (2013). Diagnosis, treatment, and outcome of giant-cell myocarditis in the era of combined immunosuppression. Circ Heart Fail.

[18] Litovsky SH, Burke AP, Virmani R (1996). Giant cell myocarditis: an entity distinct from sarcoidosis characterized by multiphasic myocyte destruction by cytotoxic T cells and histiocytic giant cells. Mod Pathol.

[19] Spence N, Niehaus K, Macias L, Cox B (2014). Giant cell myocarditis: a case report and review of the literature. Southwest J Pulm Crit Care.

[20] Kodama M, Matsumoto Y, Fujiwara M, Masani F, Izumi T, Shibata A (1990). A novel experimental model of giant cell myocarditis induced in rats by immunization with cardiac myosin fraction. Clin Immunol Immunopathol.

[21] Blauwet LA, Cooper LT (2010). Myocarditis. Prog Cardiovasc Dis.

[22] Daniels PR, Berry GJ, Tazelaar HD, Cooper LT (2000). Giant cell myocarditis as a manifestation of drug hypersensitivity. Cardiovasc Pathol.

[23] Palmer HP, Michael IE (1965). Giant-cell myocarditis with multiple organ involvement. Arch Intern Med.

